# Subcutaneous transplantation of human thyroid tissue into a pre-vascularized Cell Pouch™ device in a *Mus musculus* model: Evidence of viability and function for thyroid transplantation

**DOI:** 10.1371/journal.pone.0262345

**Published:** 2022-01-20

**Authors:** Sam M. Wiseman, Arash Memarnejadian, Guilaine K. Boyce, Anne Nguyen, Blair A. Walker, Daniel T. Holmes, Ian D. Welch, Delfina M. Mazzuca, Philip M. Toleikis

**Affiliations:** 1 Department of Surgery, St. Paul’s Hospital & University of British Columbia, Vancouver, BC, Canada; 2 Sernova Corporation, London, Ontario, Canada; 3 Department of Pathology & Laboratory Medicine, St. Paul’s Hospital, Vancouver, BC, Canada; 4 Department of Pathology & Laboratory Medicine, University of British Columbia, Vancouver, BC, Canada; Yale-New Haven Hospital, UNITED STATES

## Abstract

This study aimed to investigate the survival and efficacy indicators of human thyroid tissue transplantation into a retrievable, prevascularized implanted Sernova Corp Cell Pouch™ (CP) device. Thyroid tissue from human donors was transplanted subcutaneously into the pre-implanted CP device or into the subcutaneous (SC) space alone as a control in a nude *Mus musculus* model. Transplanted *M*. *musculus* were monitored for human serum thyroglobulin (TG) levels for 3 months until the transplants were removed for histological assessment. Human thyroid tissue survived and continued to produce TG in transplanted nude *M*. *musculus* in the CP, with no adverse events. CP transplants exhibited more persistent and robust production of human TG than tissue placed in the SC space alone from 3 to 13 weeks post transplantation. Fresh thyroid transplants had better survival and function compared to cryopreserved transplants. Thyroid transplant viability correlated with TG levels at 3 months post-transplant (p = 0.03). Immunofluorescence staining of transplants for TG and TPO localized in thyroid follicles. Human thyroid tissue transplanted into the subcutaneously implanted pre-vascularized CP in nude *M*. *musculus* survived and continued to produce robust and persistent human TG and warrants further investigation as a treatment for postoperative hypothyroidism.

## Introduction

Thyroid hormone replacement after thyroidectomy involves daily administration of levothyroxine (LT4) with dose adjustment based primarily on laboratory measurement of thyroid function (thyroid stimulating hormone (TSH), free triiodothyronine (fT3) and free thyroxine (fT4)). Despite thyroid hormone replacement being effective at compensating for gland loss, some patients may suffer from many different ongoing side effects, all reducing their overall quality of life and incurring costs by the patient and the healthcare system [1].

Interestingly, the study of thyroid transplantation (TT) as a treatment for hypothyroidism after thyroidectomy, was actually reported greater than a century ago, prior to the isolation of thyroxine [2]. Despite small preclinical and clinical studies having demonstrated its feasibility and promise, and parathyroid autotransplantation, an analogous endocrine surgical procedure, being commonly performed and well accepted world-wide, TT has only undergone very limited investigation [3,4]. This is mainly because of tolerance of life-long dependence on LT4, along with an acceptance of the associated loss of the endogenous autoregulatory mechanisms of thyroid hormone production largely due to lack of any therapeutic alternatives, that have become etched into current medical thinking and clinical dogma.

The Cell Pouch™ (CP) is a proprietary implantable and retrievable device developed by Sernova Corp. (Ontario, Canada), for the transplantation and long-term function of therapeutic cells. Upon implantation, this porous device that is composed of permanent polymer materials, becomes incorporated with highly vascularized tissue formed around a central removable polymer plug, which results in the formation of vascularized tissue chambers for the transplantation of therapeutic cells or tissue following plug removal. Approximately 4 weeks after being implanted in the subcutaneous tissues the pouch is surgically exposed, its impermeable plug is removed, and donor thyroid tissue is transplanted into the void space within the CP [5]. In a preclinical study, Pepper et al. reported that the CP is biocompatible, forms an environment suitable for pancreatic islet cell transplantation resulting in insulin independence in a diabetes model [5]. The CP is also now being studied as a treatment for type 1 diabetes in a US Phase I/II clinical trial. The current study aimed to investigate the survival and efficacy indicators of human thyroid tissue transplanted early and after a period of cryopreservation into the retrievable, subcutaneous, vascularized CP device as a potential novel approach for TT.

## Materials and methods

### Human thyroid tissue transplants

Thyroid tissue from 3 benign human goiters was procured during surgery in the practice of the study principal investigator (SMW). This study was carried out with the approval of the University of British Columbia Research Ethics Board, and all participants provided written informed consent. The patient clinical, biochemical, and pathological characteristics are summarized in Table 1. All laboratory measurements were taken preoperatively, within 4 weeks of thyroidectomy.

**Table 1:** Thyroid donor patient clinical, biochemical, and pathological characteristics.

Thyroid Donor Number	Age	Sex	Surgical Indication	Neck Radiation Exposure History	Family Thyroid Cancer History	Preop TSH (mU/L)	Preop Free T3 (pmol/L)	Preop Free T4 (pmol/L)	Postop Pathological Diagnosis
1	68	F	Goiter	No	No	0.74	4.35	12.7	Multinodular Hyperplasia
2	36	F	Goiter	Yes	No	1.06	4.42	12.8	Multinodular Goiter
3	55	M	Goiter	No	No	0.44	4.39	14.1	Multinodular Goiter

The human thyroid tissue for transplantation was procured in the operating room during thyroidectomy. A portion of the procured thyroid was immediately morcellated into 5mm^3^ pieces and aliquoted into cryovials. The remaining tissue was placed into 10% Neutral-Buffered-Formalin (NBF) for histopathological evaluation. For the immediate thyroid transplantation group, the cryovials containing 1.5ml of ice-cold Leibovitz’s L-15 transport medium (Thermo Fisher Scientific, Massachusetts, U.S.A.) with 100 μg/mL of Primocin™ (InvivoGen, San Diego, U.S.A.) were placed on ice and transported to the laboratory for transplantation in less than one hour. For the delayed transplantation group the morcellated thyroid tissue was placed into cryovials that contained 1.5ml of cryostorage medium (STEM-CELLBANKER® DMSO Free-GMP Grade, AmsBio, Massachusetts, U.S.A.) and immediately placed within an isopropyl alcohol-filled Nalgene Mr. Frosty Cryo 1˚C freezing container (Sigma-Aldrich, Ontario, Canada) on ice to allow for gradual cooling. Within 10 minutes the cryovials were transferred into a freezer for 24 hours. They were then transferred on dry-ice to the provincial Tissue Bank where they were cryopreserved for 3 months at -80˚C. On the day of transplantation, within 1 hour of being removed from the freezer, the cryopreserved thyroid tissue was transported to the transplant laboratory on dry ice.

### Animal thyroid model

**Fig 1:**
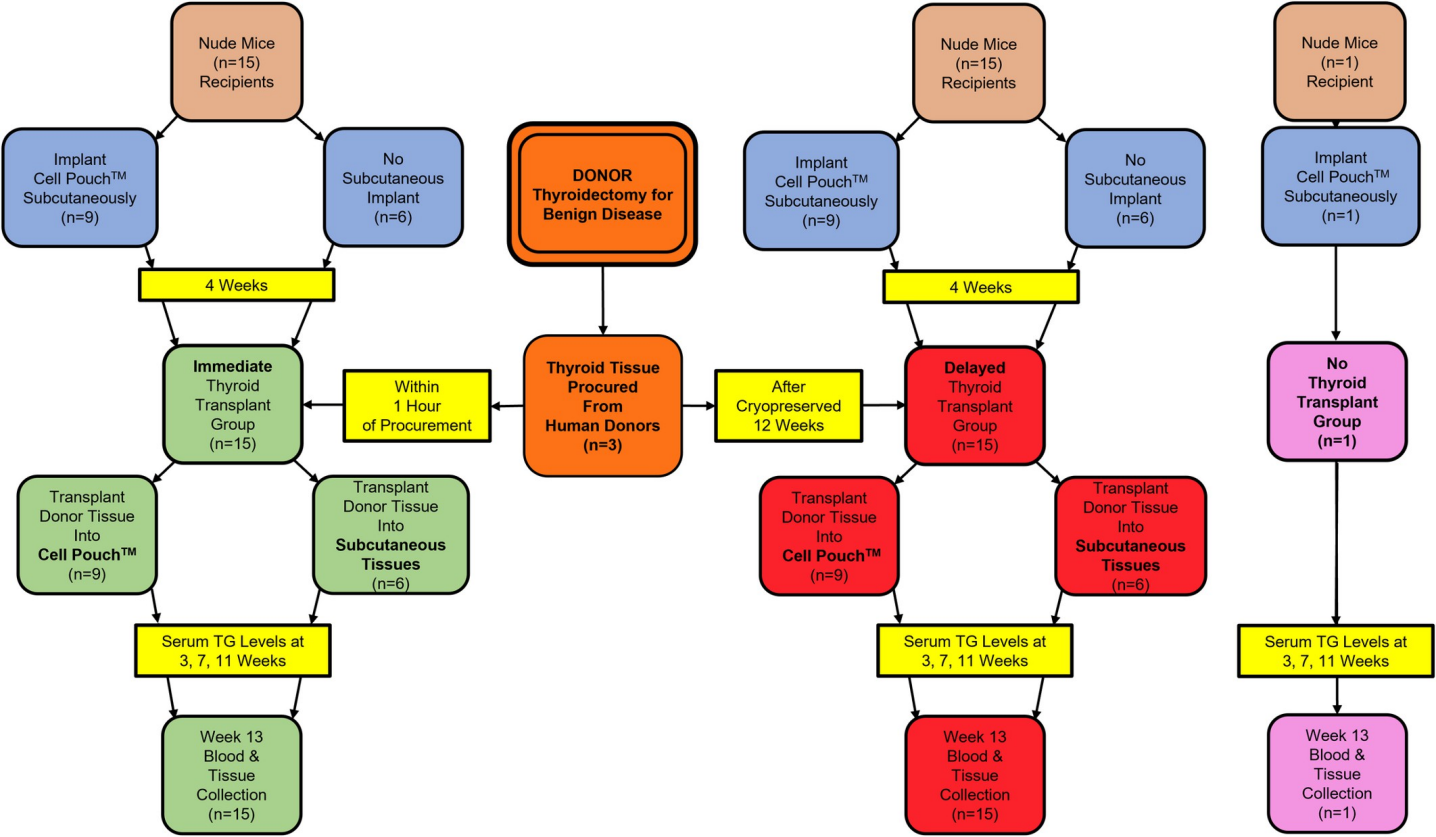
Study design. Flow diagram outlining study design and timeline.

Nude *M*. *musculus* were utilized to study TT. Female outbred euthyroid Nude *M*. *musculus* (strain Crl:NU(NCr)-*Foxn1*^*nu*^, code 490, homozygous) purchased from Charles River (Charles River, Quebec, Canada) arrived at the age of 8 weeks. As outlined in Fig 1, 18 of 31 *M*. *musculus* were implanted with the CP 1 week later. Then, 4 weeks later, all *M*. *musculus* had thyroid tissue transplanted into the previously implanted CP (18 of 31 *M*. *musculus*) or directly into the subcutaneous tissue (12 of 31 *M*. *musculus*). The *M*. *musculus* were handled and treated according to the institutional guidelines for humane animal treatment and in full compliance with relevant legislation.

### Study design and timeline

For each of the immediate and delayed transplantation groups 15 animals were utilized, such that thyroid tissue from each of the three human donors was transplanted into 5 nude *M*. *musculus* (3 into the CP that had been implanted into the subcutaneous (SC) tissues and 2 directly into the SC tissues as controls). A single *M*. *musculus* served as a negative control and was implanted with the CP but did not receive the thyroid transplant (Fig 1).

### Cell pouch implantation surgery

A one-plug Mini-Cell Pouch™ (Sernova Corporation, Ontario, Canada) was implanted subcutaneously into the abdominal wall of each of the *M*. *musculus* in the CP groups 4 weeks prior to thyroid tissue transplantation to enable tissue incorporation into the device. After the *M*. *musculus* was anesthetized, its abdominal wall was disinfected and a transverse ~5mm incision was made in the skin just lateral to the midline. A subcutaneous pocket was then created caudal to the incision and a 6mm x 15mm CP was placed inside it, such that the opening of the pouch was oriented cranially, and its plug was positioned parallel to the midline. The CP was then sutured from the top of its chamber to the underlying abdominal wall muscle. The incision was then sutured closed. Animals were then recovered and monitored for 3 days.

### Thyroid transplantation surgery

For the immediate transplantation group, the thyroid transplants were performed within 1 hour of procurement. For the delayed transplantation group the cryopreserved thyroid tissue was thawed immediately prior to transplantation. For tissue thawing, the vial containing the tissue was alcohol disinfected, and while the tissue was still on ice, 100 μL of room temperature transport medium was added. The tissues were then washed with 9 mL of Primocin™-containing Leibovitz’s L-15 medium to remove the freezing medium, prior to transplantation into the *M*. *musculus*.

**Fig 2:**
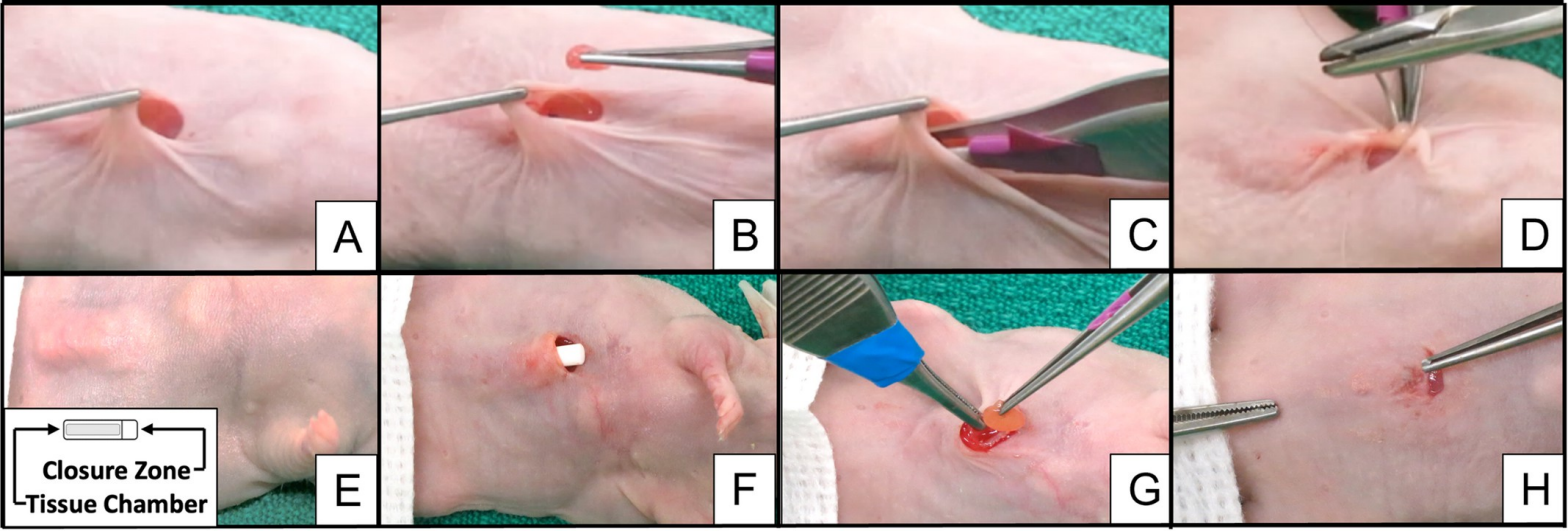
Transplantation procedure. Transplantation of morcellated human thyroid tissue into the nude *M*. *musculus* abdominal wall subcutaneous tissues (**A-D**) or subcutaneous Cell Pouch™ that was implanted 4 weeks earlier (**E-H**). Image inset in **E** shows schematic of subcutaneous Cell Pouch™.

The transplanted *M*. *musculus* was anesthetized and its abdominal wall skin was disinfected. For the CP implant group, a 3mm transverse incision was made laterally to the midline, just cranially to the top of the CP that was easily palpable in the subcutaneous tissues. A transverse incision was then made in the tissue just above the CP to expose its closure zone. The closure zone was opened, and the plug removed exposing the vascularized tissue chamber void space for tissue transplantation. For the SC transplants a pocket was created in the subcutaneous tissues in a similar location and size as the implant in the CP group. Meanwhile, the thyroid transplant tissue was cut into 2mm x 3 mm pieces and weighed. The maximum amount of tissue that filled a 1-plug CP was 0.1g. Animals were transplanted with 4–6 pieces (0.05g - 0.1g) of thyroid tissue either into the subcutaneous CP, or into a SC pocket. For the *M*. *musculus* with SC transplants the site of transplantation was marked by two distal and proximal sutures on the adjacent deep musculature using a violet 6–0 Prolene suture. For the *M*. *musculus* with the CP implants, after the thyroid tissue was loaded into its chamber, the pouch’s opening was sutured closed. The skin incision was then sutured closed, and the animals were recovered and monitored (Fig 2).

### Thyroid tissue pre-transplantation viability assessment

The MTT viability assay, a colorimetric assay for assessing cellular metabolic activity, was used to evaluate both the fresh and cryopreserved thyroid tissue viability immediately prior to it being transplanted into the *M*. *musculus*. The optical density of transplant specimens was divided by their dry tissue weight to calculate the weight-corrected OD (wc-OD). The assay was carried out in triplicate and the mean wc-OD for each thyroid transplant was calculated [6].

### Serum human thyroglobulin level measurement

In accordance with the approved institutional Animal Use Protocol, we collected up to 15% of the circulating blood volume from the saphenous vein of the *M*. *musculus* (72 mL/Kg) every 4-weeks starting 1 week prior to transplantation. At necropsy, a terminal bleeding from the inferior vena cava was performed. Blood samples were collected in microfuge tubes, stored at room temperature, and allowed to completely coagulate, and then centrifuged at 2500g for 15 minutes. The serum was then isolated, aliquoted and stored at -20 ˚C, and later at -80 ˚C, until analyzed. Thyroid function tests on *M*. *musculus* serum samples were performed on the Roche Cobas e601 immunoassay analyzer (Roche Diagnostics, Quebec, Canada) using a competitive electrochemiluminescent immunoassay for fT4 and an electrochemiluminescent immunometric (“sandwich”) assay for TG. The fT4 immunoassay used was the Cobas FT4 II, which has an analytical measuring range of 0.3 to 100 pmol/L and observed analytical coefficients of variation (CVs) of 1.4% at 10.9 pmol/L and 1.6% at 22.0 pmol/L. The TG immunoassay used was the Cobas Tg II assay, which has an analytical measuring range of 0.04 to 500 ng/mL above which concentration dilutions must be performed. Our observed CVs for this assay range from 2.3–5.1% for TG concentrations ranging from 0.9–53 ng/mL.

To ensure that the Roche Cobas assay demonstrated no immunoreactivity to *M*. *musculus* TG but was capable of returning free hormone results (despite necessarily dissimilar binding globulins), pooled serum from three *M*. *musculus* was analyzed for TG, antithyroglobulin antibody, free triiodothyronine (fT3) and free thyroxine (fT4). Protein assays returned undetectable results of <0.04 ng/mL and <10 kIU/L, respectively, while the pool returned a fT3 value of 4.5 pmol/L and a fT4 value of 31.7 pmol/L. While there is no guarantee of the accuracy for the free hormone measurements in *M*. *musculus*, it did demonstrate that there was a measurable signal. Likewise, in *M*. *musculus* with intact thyroid glands, no immunoreactivity for TG was detected, suggesting that any signal obtained from transplanted *M*. *musculus* was attributable to the presence of human TG.

### Thyroid transplant histology & immunohistochemistry

At the study endpoints (3 months post-transplantation), animals in both the immediate and delayed thyroid transplantation groups were sacrificed for necropsy and blood/tissue collection. The thyroid transplants from both the CP and SC groups were recovered and fixed in 10% Neutral-Buffered Formalin (NBF) for 48 hrs, washed with Phosphate-Buffered Saline (PBS), and submerged in 70% ethanol. Specimens were then dehydrated in an Excelsior AS automated tissue processor (Thermo Fisher Scientific, Massachusetts, U.S.A.) using the “Routine Overnight Program”, paraffin embedded in blocks and sequentially cut into 5 μm sections for routine H&E staining. Immunofluorescence staining of the thyroid transplants with anti-human thyroglobulin (TG) (cat #ab92467, AbCam, Cambridge, U.K.) and anti-human thyroperoxidase (TPO) (cat #NBP2-27544, Novus Biologicals, Colorado, U.S.A) antibodies was carried out in accordance with manufacturer instructions with recommended positive and negative controls. Native *M*. *musculus* thyroid tissue was also stained but did not label with these antibodies.

### Statistical analysis

The differences in thyroid transplant viability and *M*. *musculus* serum human TG levels for the CP and SC transplants, and the immediate and delayed transplantation groups, were evaluated using the paired two-tailed student’s t-test. The correlation between the TG levels and the thyroid tissue viability was performed using linear regression analysis. The fisher’s test was used to evaluate the proportion difference between the viability of the CP and SC transplant groups. The threshold for significance (alpha) was set to 0.05 in all analysis. Ninety-five percent confidence intervals are calculated and displayed in the Figs. There was no modification of outliers in the dataset for the statistical analysis. Statistical analysis was performed using R version 3.6.0. The coding required for this analysis is openly available at https://dataverse.scholarsportal.info/privateurl.xhtml?token=ebbc9127-e056-4fc3-bc44-d0caf3b477f5.

## Results

The study data are openly available in the University of British Columbia research collection of the Scholars Portal Dataverse at: https://dataverse.scholarsportal.info/privateurl.xhtml?token=ebbc9127-e056-4fc3-bc44-d0caf3b477f5. This study was carried out in strict accordance with the ’Principles of Laboratory animal care’ NIH publication Vol 25, No. 28 revised 1996; http://grants.nih.gov/grants/guide/noticefiles/not96-208.html) and Canadian laws. The protocol was approved by the University of British Columbia Animal Care Committee (Protocol Number: A16-0296). All surgery was performed under 5% isoflurane anesthesia. During the study procedures every effort was made to minimize suffering.

### Thyroid transplant donor characteristics

The clinical, biochemical, and pathological characteristics of the 3 thyroid tissue donor patients is summarized in Table 1. All patients were euthyroid preoperatively and their preoperative diagnosis was benign goiter, which was confirmed by postoperative pathological evaluation. Donor 2 had a history of receiving 15 mCi of radioactive iodine 1 year prior to thyroidectomy for treatment of subclinical hyperthyroidism, with subsequent normalization of thyroid function. The mean weight of transplanted thyroid tissue from donors 1, 2, and 3 was 0.10 g, 0.10 g and 0.07 g, respectively.

### Thyroid pre-transplant viability

**Fig 3:**
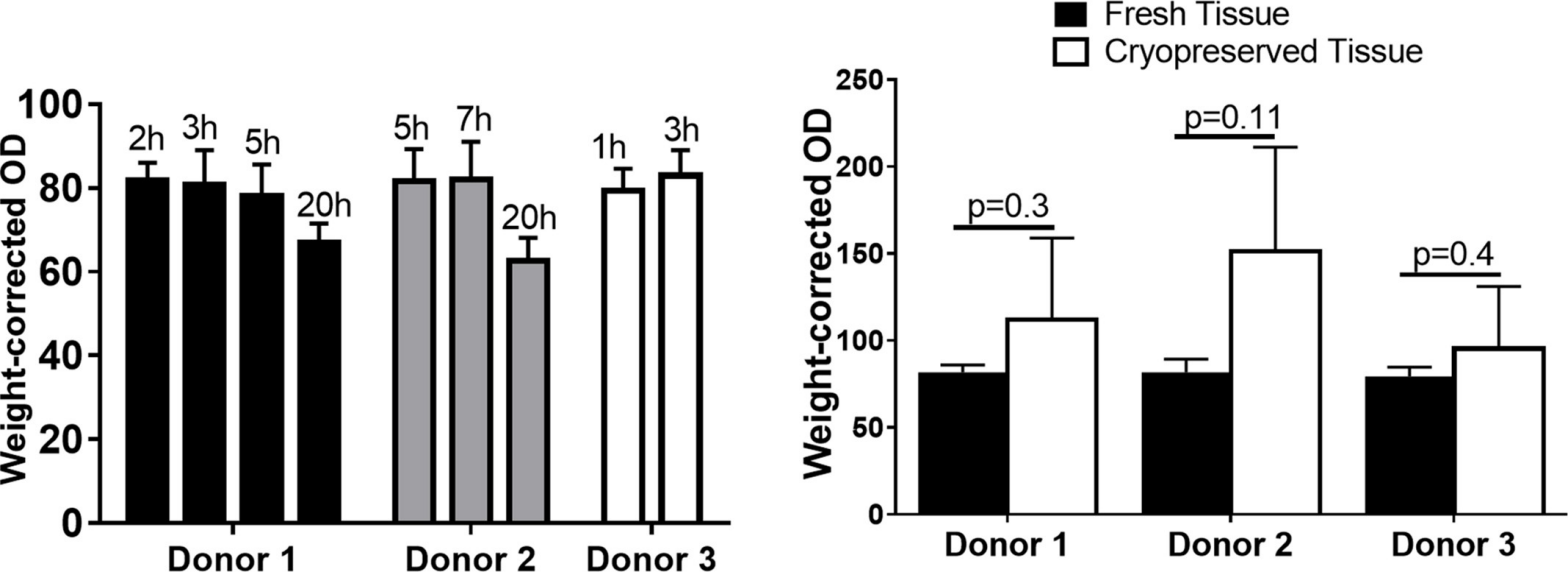
Pre-transplantation viability assessment. Thyroid tissue viability assay. Human thyroid tissue specimens procured from 3 donors were evaluated immediately prior to transplantation with a tissue MTT viability assay. (A) Immediate transplants (fresh thyroid tissues stored on ice since procurement for different time periods of 1, 2, 3, 5, 7 and 20 hours) were tested. (B) Immediate (fresh) and delayed (cryopreserved and thawed) tissues from each individual donor were assayed for their viability before transplantation and data were compared using student’s t-test. Error bars show Mean ± SD.

As shown in Fig 3 the MTT tissue viability assay demonstrated that the immediate and delayed thyroid transplants showed no significant difference in viability prior to transplantation.

### Histopathological evaluation of thyroid transplants

**Fig 4:**
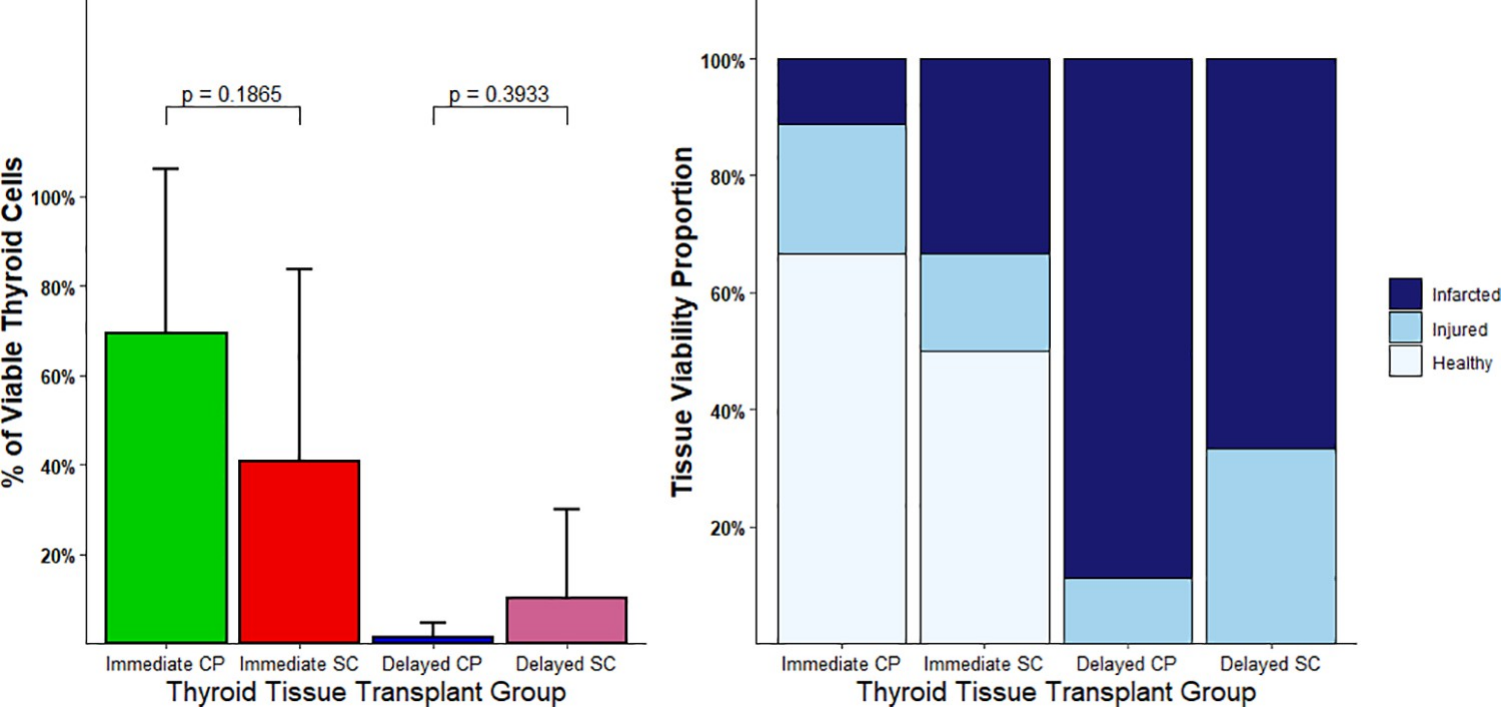
Post-transplantation viability. **A.** Percentage of viable thyroid cells present in a 2mm diameter microscopic field for the immediate and delayed thyroid transplantation groups for both the Cell Pouch^TM^ (CP) and subcutaneous tissue (SC) transplants. **B.** Tissue viability of the immediate and delayed thyroid transplantation groups for both the subcutaneous CP and the SC control thyroid transplants. Viable thyroid tissue (light blue), injured/unhealthy thyroid tissue (medium blue) and no viable thyroid tissue (dark blue). Error bars show Mean ± SD.

The thyroid transplants retrieved after animal sacrifice were evaluated for cellular viability by a pathologist who scored H&E-stained sections for the percentage of viable thyroid cells (Fig 4A). For the immediate transplantation group the mean differences between the percentage of viable cells for the CP and SC transplants were calculated. Even though the thyroid tissue that was transplanted into the CP had a 28.4% absolute increase in the percentage of viable cells relative to the SC transplants, this result did not reach statistical significance (SD = 24.9, p = 0.18). However, the viability of the transplanted cells within the CP in the immediate transplantation group compared to the delayed transplantation group was significantly increased by an absolute value of 66.4% (SD = 11.9, p = 0.0094). Overall, these observations suggest that human thyroid transplants within the CP have similar survival to the SC tissue control; however, there is a trend towards improved survival within the CP. In addition, the transplants had significantly improved survival when transplanted early compared to after 3 months of cryopreservation.

The H&E slides of the thyroid transplants were also evaluated by a pathologist for overall tissue viability and categorized into a 3-tier categorical qualitative scale: no viable thyroid tissue, presence of injured/unhealthy thyroid tissue, and presence of viable thyroid tissue. As shown in Fig 4B, this assessment found that the immediate transplantation groups had healthier appearing thyroid tissue than the delayed transplantation groups. In the immediate thyroid transplantation group the proportion of *M*. *musculus* with healthy thyroid transplants was 67% in the CP transplantation group compared with 50% in the SC transplantation group (OR 3.62, p = 0.52). There was no pathological evidence of thyroid malignancy present in any of the thyroid transplants, or the native mouse thyroid glands, which were also evaluated.

### Immunohistochemical evaluation of thyroid transplants

**Fig 5:**
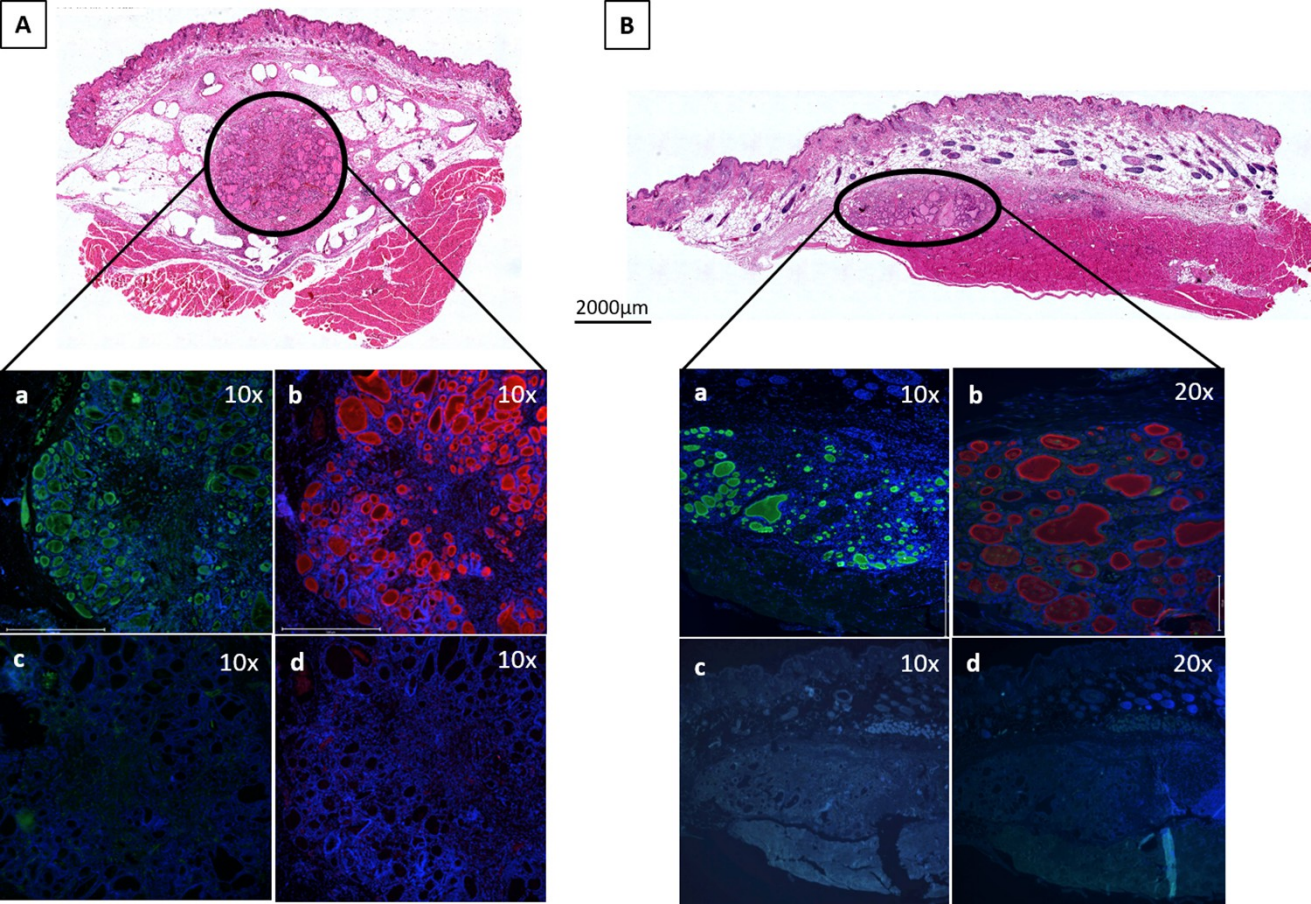
Immunohistochemistry of thyroid tissue transplants. Images of stained thyroid transplants from both the (A) Cell Pouch™ and (B) Subcutaneous Tissues immediate transplantation group. Transverse 5μm sections from both specimens were stained with H&E (upper images) as well as (a) anti-human TG and (b) anti-human TPO. Thyroid follicles containing TG and TPO stained green and red, respectively. DAPI was used for nuclear counterstaining (blue). Negative control images of (c) TG and (d) TPO staining, lacking the primary antibodies, were taken with equivalent microscope setting and are shown below the related images. Magnification is indicated in the upper right corner of images.

Representative transplant sections from both the CP and SC immediate transplant groups that had a high cellular and tissue viability by histology at 13 weeks post-transplantation were further evaluated by immunofluorescence staining for human TG and human TPO expression. As shown in Fig 5 for these cases, the thyroid follicles from both the CP and SC tissue transplants stained strongly for both human thyroid-specific markers. Negative control counterparts that were processed in the absence of primary antibodies and imaged with equivalent microscope settings did not stain for these markers.

### *Mus musculus* serum human thyroglobulin measurement

**Fig 6:**
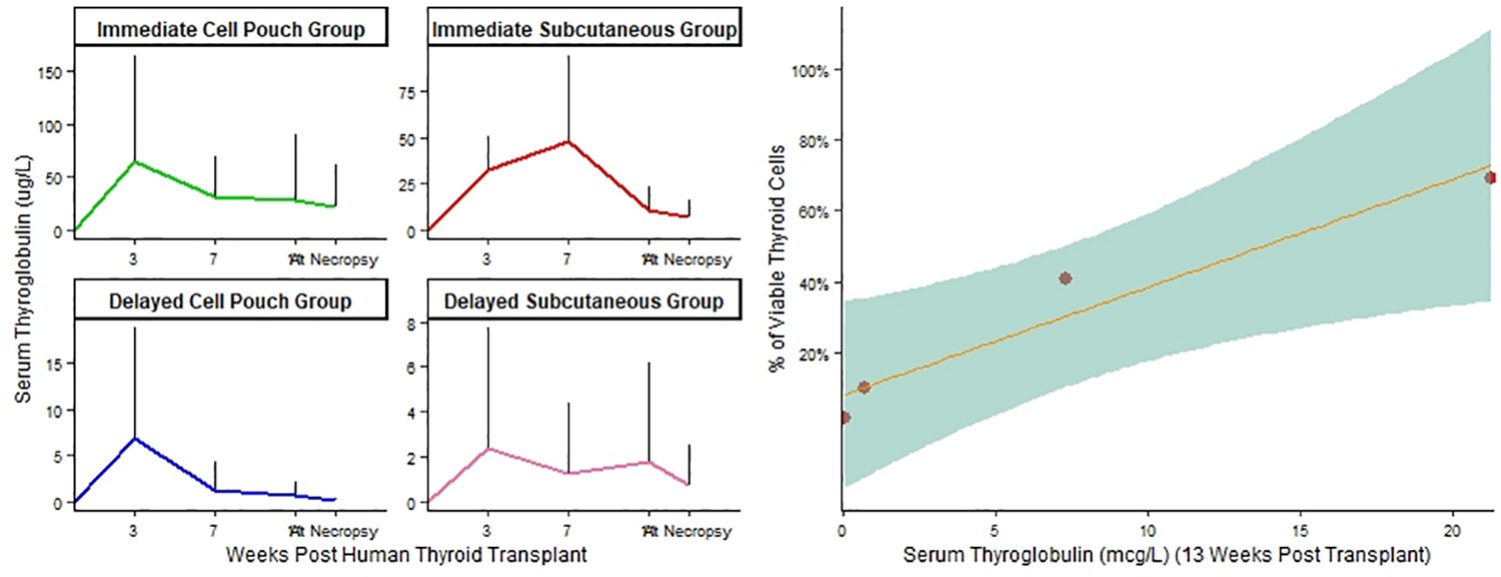
**A.** Human thyroglobulin levels. Average human thyroglobulin levels measured over time for the immediate transplantation group (green indicates thyroid transplanted into subcutaneous Cell Pouch^TM^ (CP) and red indicates thyroid transplanted into subcutaneous tissue (SC)) and delayed transplantation group (blue indicates thyroid transplanted into subcutaneous CP and violet indicates thyroid transplanted into SC). The error bars represent standard deviation. Each study group was composed of 15 *M*. *musculus*. There was no statistical difference in the TG levels in the immediate CP transplantation group when compared to the immediate SC transplantation group (SD = 37.4, p = 0.60). Variation in weight of the transplanted thyroid tissue contributed to TG level variance in all study groups. **B.** The human average serum thyroglobulin level at 13 weeks post-transplantation and thyroid transplant viability in the immediate CP transplant group (percent of viable thyroid cells in a 2mm microscopic field) were strongly correlated (R^2^ = 0.94, p = 0.03).

Circulating human TG was measured in both the immediate and delayed thyroid transplantation *M*. *musculus* groups over time, and these results are shown in Fig 6A. Immediately prior to sacrifice at week 13 post-transplant only the immediate thyroid transplantation group had measurable average serum TG levels. These TG levels were 21.3 ug/L in the CP transplant group and 7.3 ug/L in the SC transplant group. As shown in Fig 6A, the average human TG levels in the immediate CP transplantation group were more persistent across the 4 time periods over which they were measured, when compared to the immediate SC transplantation group, which demonstrated a steady decline in TG between weeks 7 and 13 (p = 0.0001). Despite there being no statistically significant differences in TG levels when comparing the immediate CP and SC groups for any specific study time period (difference in TG levels at week 13, SD = 37.4, p = 0.60) or in total (p = 0.36), a subgroup analysis of the histologically healthy thyroid tissue cases revealed that the immediate CP group total TG levels were significantly higher than the immediate SC group total TG level (p = 0.0067). These observations suggest that while human TG continues to be produced by both the CP and SC thyroid transplants for longer than 3 months post-transplantation, among the surviving transplants (healthy tissue present at week 13 post-transplantation), the CP transplants exhibited more persistent and more robust production of human TG. As shown in Fig 6B the average human serum TG level measured at week 13 post-transplant strongly correlated with the average percentage of viable thyroid cells present in a 2mm diameter microscopic field (R^2^ = 0.94, p = 0.03).

## Discussion

Hypothyroidism is the predictable consequence of total thyroidectomy, and after hemithyroidectomy an estimated 20% of patients will also eventually go on to become hypothyroid [7]. Both national and international guidelines on the management of hypothyroidism currently recommend LT4 monotherapy, given at a dose that normalizes the serum TSH, as its treatment of choice [8–11]. Though thyroxine (T4) does have intrinsic activity, many tissues have deiodinases that activate T4 to triiodothyronine (T3), which is the significantly more biologically active thyroid hormone [1]. Achieving a normal serum TSH level in patients receiving LT4 monotherapy may be complex because it is impacted by many different factors that include: compliance, consistency of clinical monitoring, food intake, medications, renal T4 loss, gastrointestinal malabsorption, pregnancy, body weight, sex, age and even the presence of antibodies that interfere with TSH measurement [12]. Even for those on LT4 monotherapy with TSH normalization approximately 5–10% of biochemically euthyroid individuals report symptoms that include lethargy, fatigue, memory impairment, depression, cold intolerance, hoarseness, dry skin, body weight gain and constipation [1]. One possible reason for persistent symptoms is differences in the inter-individual hypothalamic-pituitary-thyroid axis ‘set-point’ for circulating thyroid hormone concentrations. Another possible explanation is based on the observation that for individuals with an intact thyroid gland approximately 20% of their circulating T3 comes from intrathyroidal deiodination of T4 and/or direct thyroidal secretion, and 80% comes from peripheral T4 conversion by deiodinases. This is not the case for hypothyroid individuals on LT4 monotherapy in whom all T3 is derived from peripheral LT4 conversion [1]. Thus, compared to individuals with an intact thyroid gland, some people receiving LT4 monotherapy will have lower circulating free T3 to T4 ratios, and some may not even maintain normal serum T3 or T4 levels [1,13]. In an attempt to improve patient outcomes by increasing the serum T3 level, combination T3 and T4 therapy has been suggested. Interestingly, more than a dozen clinical trials have failed to clearly demonstrate superiority of T3 and T4 combination therapy over T4 monotherapy alone for treatment of hypothyroidism [14].

The TT literature is quite heterogeneous and generally considered controversial because it is primarily composed of historical reports, experimental animal studies and small case series [3]. This literature is extremely inconsistent in terms of the preoperative transplant thyroid pathology, whether fresh or cryopreserved tissue is transplanted, the transplant volume, the anatomical site for transplantation, the protocols for transplant functional evaluation and monitoring, and virtually all other procedural details [3]. Our approach to TT, in which thyroid tissue is transplanted into an implanted prevascularized medical device, is unique and may have clinical benefit over other methods. The CP has many characteristics that make it more attractive for TT than direct transplantation into recipient tissues in humans and these include simplicity and ease of accessibility for placement or retrieval from the subcutaneous tissues, biocompatibility, dosability, scalability and safety [5]. Also because the CP leads to the development of a pre-vascularized transplant recipient environment, it could potentially facilitate better thyroid transplant survival and function that results in finer control over tissue dosing.

Despite the MTT viability assay suggesting that there was good survival of both the procured fresh and cryopreserved thyroid tissue immediately prior to transplantation, we did observe significantly reduced cellular and tissue viability, as well as TG production, from the cryopreserved thyroid transplants. This suggests that further study and optimization of the thyroid cryopreservation protocols is needed, as there are many possible clinical scenarios where delayed transplantation could be advantageous. This would include banking thyroid tissue post-hemithyroidectomy for possible future transplantation to treat hypothyroidism or delaying transplantation until after final pathological evaluation for diagnostic thyroid operations to avoid inadvertent transplantation of malignancy. The strong correlation between the average serum TG levels measured at 13 weeks post-transplant and transplant cellular viability, along with the rapid disappearance of the human TG from the serum of the *M*. *musculus* in the delayed transplantation group that had poor transplant survival. These results along with the observed human TG staining of viable thyroid follicles, together suggests that TG was being produced by the CP thyroid transplant in a manner similar to the control SC thyroid transplants. While this approach could be beneficial in the clinic to avoid post-thyroidectomy hypothyroidism, clinical TT protocol details must first be established. These include, but are not limited to, defining the clinical settings where TT is most appropriately applied (total versus hemithyroidectomy and specifics regarding underlying thyroid pathology, and in other clinical settings when the thyroid gland is removed such as during larynx surgery), details regarding TT technique (immediate versus delayed) and dose scaling from preclinical to the patient. Local Immune protection of thyroid tissue transplanted into the CP also represents a novel future strategy that would facilitate allotransplantation and allow for treatment of the nonsurgical hypothyroidism patient population. The CP could also potentially serve as a component of a bioartifical thyroid gland. Such approaches could incorporate functional thyroid epithelia differentiated from pluripotent stem cells, or bioprinting using embryonic thyroid tissue spheroids, both of which show early promise and have been reported in preclinical studies [15,16].

The observations made in the current study have several limitations that include the small number of human thyroid tissue donors and transplants that were evaluated, along with a limited follow up period. Development of clinical or subclinical hypothyroidism in the immediate preoperative period could impact results, and so in future TT study protocols measurement of thyroid function and thyroperoxidase antibodies (TPO) at the time of surgery should be included. Even though the observations made in this study indicate that human thyroid tissue transplanted into the subcutaneously implanted pre-vascularized CP survives and continues to produce human TG that is measurable in the serum after 13 weeks, and is present in thyroid follicles with viable histology, it cannot be inferred that the transplants are producing fT3 or fT4 and restoring thyroid autoregulation. The production of fT3 and fT4 from TG is a complex multistep process [17]. As the nude *M*. *musculus* we evaluated had intact thyroid glands, and human and *M*. *musculus* fT3 and fT4 were not distinguishable in the *M*. *musculus* serum, additional study in a thyroidectomized animal model could provide further support in evaluating efficacy. In conclusion, our CP based approach to TT warrants further investigation as a treatment for postoperative hypothyroidism as it could potentially allow for avoidance of the requirement for life long LT4 monotherapy and its many associated shortcomings.
